# A Rare Case of Urogenital Myiasis in a 49-Year-Old Woman

**DOI:** 10.1155/2022/7910176

**Published:** 2022-04-01

**Authors:** Amir Mohammad Salehi, Ensiyeh Jenabi, Maral Salehi

**Affiliations:** ^1^Medical Student, School of Medicine, Hamadan University of Medical Sciences, Hamadan, Iran; ^2^Assistant Professor of Mother and Child Health, Autism Spectrum Disorders Research Center, Hamadan University of Medical Sciences, Hamadan, Iran; ^3^Obstetrics and Gynecology Ward, Valiasr Hospital, Birjand University of Medical Sciences, Birjand, Iran

## Abstract

Myiasis refers to the infestation of living vertebrae with fly larvae, principally occurring in individuals with a low socioeconomic status and poor personal hygiene. Myiasis is rarely manifested in the urogenital system. Herein, we report a case of urogenital myiasis in a 49-year-old rural woman complaining of maggots in the urine and severe genital itching.

## 1. Introduction

The term myiasis is derived from the Greek root mya meaning flight and was coined by Hope FW in 1840 when describing the infestation of living vertebrae with dipterous larvae [1]. This condition involves the infestation of living vertebrae with dipterous maggot larvae [2]. Human myiasis (HM) is globally reported and is mostly prevalent among families with a poor socioeconomic status residing in tropical regions. It is, however, not limited to these regions and manifests as a global agricultural concern when occurring in domestic animals. In humans, this condition is usually induced by parasites related to an animal host. Cutaneous myiasis is the most prevalent clinical form of the condition [2]. Nasopharyngeal myiasis involves the infection of the nose, mouth, sinuses, and ears. Other forms include ocular myiasis (ophthalmomyiasis) and intestinal myiasis due to the ingestion of the organism. Vaginal and urogenital myiasis is rarely reported [3]. Herein, we present a case of urogenital myiasis in a 49-year-old woman.

## 2. Case Report

A 49-year-old rural woman presented three days ago to our Charity Clinic, complaining of maggots in the urine and severe genital itching. Her family occupation was farming and animal (sheep, goats, and cows) husbandry, and they had a relatively low economic status.

She reported foul-smelling vaginal discharge for the past six months but had not visited the clinic due to fear of social stigma. She reported no history of underlying diseases, trauma, or insect bite to the vaginal area. She did not smoke cigarettes but used hookah. In the past six months, the patient did not have sexual intercourse and had regular menstruation.

**Figure 1:**
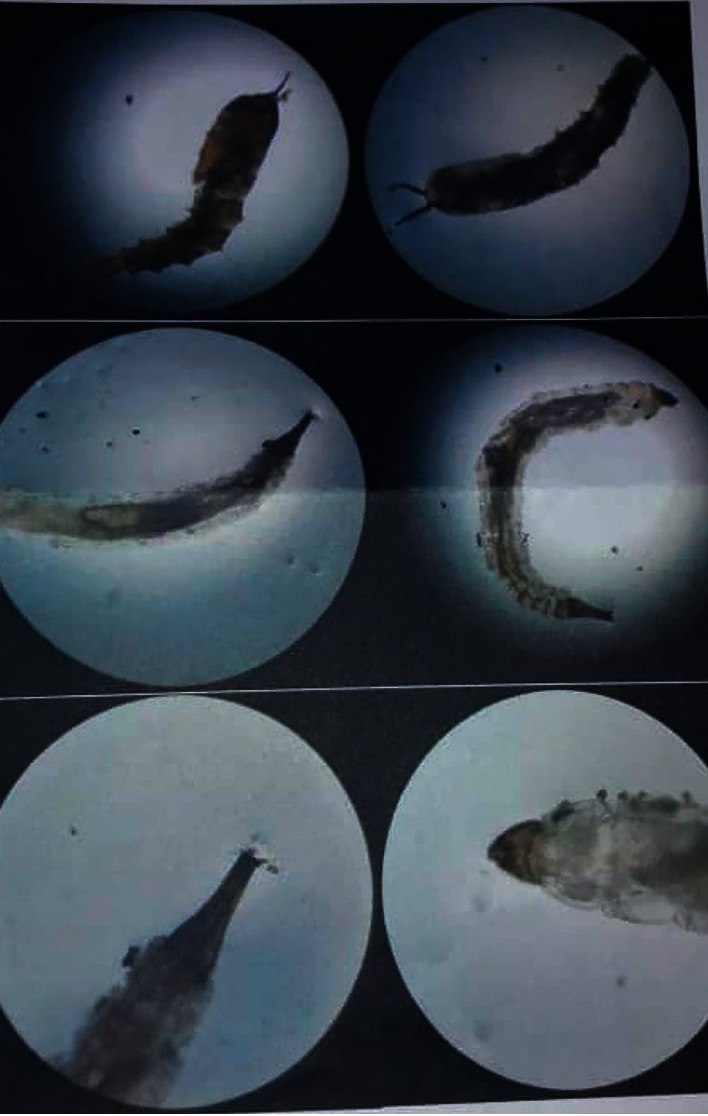
Urine analysis reported a large number of larvae (a maximum size of 4 mm) without RBC or WBC. The morphology of the larvae was compatible with flies.

She reported wearing underpants and skirts. The physical exam revealed erythema in the labia major and around the groins due to severe itching. In the vaginal exam, inflammation and a large number of maggots were observed in the urethral meatus, labia minora, and vaginal canal, progressing to near the cervix. Still, the cervix seemed intact on colposcopy. Infection and necrosis were observed in the labia minora due to the existence of larvae that ate away at the tissues. No significant lymphadenopathy was observed. Many maggots were sampled for microscopical examination. About 50-60 maggots were separated from the labia minora and about 10 from the vaginal canal. Blood (WBC: 9.2/mm^3^, Hgb: 14gr/dl) and stool test results were normal, but urine analysis reported a large number of larvae (a maximum size of 4 mm) without RBC or WBC. The morphology of the larvae was compatible with flies (family Insect) (Figure 1). The patient could not afford an entomological examination of the parasite. Cystoscopy was requested to examine bladder and urinary tract infection. Cystoscopy revealed gross erosion and burrowing of the left lateral urethral wall, and large clots embedded with maggots were identified within the bladder lumen.

The bladder was quickly rinsed, and a Foley catheter was placed for one week. About 30 maggots were removed after rinsing the urinary tract. To remove the maggots possibly existing deep into the vaginal tissue, 15% chloroform in paraffin oil was used to rinse the patient's vagina for 48 hours. The infected and necrotic parts of the labia minor were debrided. To prevent reinfection and secondary bacteremia, the patient was treated with amoxicillin, clavulanic acid (1.2 g twice a day), and metronidazole (500 mg three times a day) for one week. She was admitted to the gynecology ward for one week. Upon discharge, she was recommended to keep her vagina and living environment clean and not to wear skirts when working at the farm or with animals. Two weeks later, cystoscopy results were normal, the infected parts of the labia minora were improved, and no maggots were found in the vaginal exam.

## 3. Discussion

Myiasis is an ectoparasitic infection of humans and vertebrae, the most prevalent form of which is cutaneous myiasis. Nasopharyngeal myiasis involves the infection of the nose, mouth, sinuses, and ears. Other forms of this condition include ophthalmomyiasis, intestinal myiasis, and urogenital myiasis [4, 5]. Infestation with larvae can be caused by obligate or facultative parasites. When dipterous larvae enter the human tissue, they follow three stages of growth and feeding, after which they transform into pupa and then to mature flies [3, 6]. *Cochliomyia hominivorax, Chrysomya bezziana,* and *W. magnifica* are the most prevalent flies causing obligatory human wounds worldwide [7].

Urogenital myiasis constitutes only 0.7% of human infestations, with very few cases reported so far [3, 8]. A recent systematic review found the vagina as the most prevalent site of female urogenital myiasis [9]. In our case, myiasis involved the labia minora, urinary bladder, urethral meatus, and vaginal canal. Therefore, the flies may lay their eggs near the urethra, and then, the larvae hatch and migrate to the bladder.

The risk factors of external urogenital myiasis include a low socioeconomic status, inappropriate health conditions, poor mental and physical health, a lack of personal hygiene, cervical cancer, urinary tract stents, and sexually transmitted infections [10].

Treatment aims to clean the tissue from larvae. Sometimes, manual pressure is enough for cutaneous myiasis, which is usually performed before visiting the doctor. Surgery is almost always indicated for the migratory form of the disease. The general myiasis management protocol includes the mechanical removal of all the visible larvae, followed by debridement and, occasionally, rinsing with disinfectant solutions and bandaging, until all the maggots are removed [3]. The use of 15% chloroform in olive or other oils, or even ether, demobilizes the larvae. In our patient, the vagina was rinsed with 15% chloroform in liquid paraffin for 2 days.

Myiasis can be prevented by using DEET (chemical name, N,N-diethyl-meta-toluamide) containing pesticides and mosquito nets. Ironing is an effective method for killing the eggs. Other preventive measures include wearing long-sleeved clothes to cover the wounds and not sleeping outdoors [11].

## Data Availability

The data used to support the findings of this study are available from the corresponding author upon request.
